# Jinlida Granules Reduce Obesity in db/db Mice by Activating Beige Adipocytes

**DOI:** 10.1155/2022/4483009

**Published:** 2022-05-19

**Authors:** Hong-ru Zhou, Tong-xing Wang, Yuan-yuan Hao, Yun-long Hou, Cong Wei, Bing Yao, Xuan Wu, Dan Huang, Hui Zhang, Yi-ling Wu

**Affiliations:** ^1^Hebei Medical University, No. 361 Zhongshan Road, Chang'an District, Shijiazhuang, Hebei Province, China; ^2^National Key Laboratory of Collateral Disease Research and Innovative Chinese Medicine, Shijiazhuang, China; ^3^Key Laboratory of State Administration of TCM (Cardio-Cerebral Vessel Collateral Diseases), Shijiazhuang, China; ^4^Hebei University of Chinese Medicine, Shijiazhuang, China; ^5^Affiliated Hospital of Integrated Traditional Chinese and Western Medicine, Nanjing University of Chinese Medicine, Nanjing 210028, China; ^6^The First Affiliated Hospital of Henan University of CM, Zhengzhou, China

## Abstract

Recent studies indicate existence of beige adipocytes in adults. Upon activation, beige adipocytes burn energy for thermogenesis and contribute to regulation of energy balance. In this study, we have analyzed whether Jinlida granules (JLD) could activate beige adipocytes. JLD suspended in 0.5% carboxymethyl cellulose (CMC) was gavage fed to db/db mice at a daily dose of 3.8 g/kg. After 10 weeks, body weight, biochemical, and histological analyses were performed. In situ hybridization, immunofluorescence, and western blotting were conducted to test beige adipocyte activation in mice. X9 cells were induced with induction medium and maintenance medium containing 400 *μ*g/mL of JLD. After completion of induction, cells were analyzed by Nile red staining, time polymerase chain reaction (PCR), western blotting, and immunofluorescence to understand the effect of JLD on the activation of beige adipocytes. A molecular docking method was used to preliminarily identify compounds in JLD, which hold the potential activation effect on uncoupling protein 1 (UCP1). JLD treatment significantly improved obesity in db/db mice. Biochemical results showed that JLD reduced blood glucose (GLU), triglyceride (TG), and low-density lipoprotein cholesterol (LDL) levels as well as liver aspartate aminotransferase (AST) and alanine aminotransferase (ALT) levels in mice. Hematoxylin and eosin staining (H&E) showed that JLD reduced hepatocyte ballooning changes in the liver. Immunofluorescence showed that JLD increased the expression of the thermogenic protein, UCP1, in the beige adipose tissue of mice. JLD also increased the expression of UCP1 and inhibited the expression of miR-27a in X9 cells. Molecular docking results showed that epmedin B, epmedin C, icariin, puerarin, and salvianolic acid B had potential activation effects on UCP1. The results suggest that JLD may activate beige adipocytes by inhibiting miR-27a expression, thereby promoting thermogenesis in beige adipocytes. This study provides a new pharmacological basis for the clinical use of JLD.

## 1. Introduction

The prevalence of obesity is increasing globally. Assuming an annual growth rate of 2.6%, the number of obese adults in 2025 will be 40% higher than that reported in 2012 [1]. The most striking feature of obesity is the abnormal adipocyte proliferation and hypertrophy. Hypertrophic adipose tissue secretes adipokines that stimulate various organs and cause diseases [2] such as metabolic syndrome, coronary heart disease, diabetes, and nonalcoholic steatohepatitis [3–6]. However, treatment options for obesity are currently limited.

Beige adipocytes are regarded as the third type of adipocyte with an intermediate morphology between white and brown adipocytes (BAT). Although like brown adipocytes in the thermogenic function [7], beige adipocytes sporadically reside in the areas of white adipose tissue (WAT) depots rather than in the BAT depots. In addition, current research suggests that BAT are derived from myf5+ progenitor cells [8], while beige adipocytes may originate from precursors of white adipocytes [9]. In the inactive state, beige adipocytes resemble WAT with an extremely low basal expression of UCP1 but are stimulated by cold exposure, receptor activation, and exercise, the expression of UCP1 will be regulated, and its function is changed as same as that of classic BAT. Thus, to some extent, beige adipocytes are considered the inducible BAT. Due to the thermogenic function, activated beige adipocytes can alleviate the disorders of blood glucose and lipid metabolism in mice [10]. Although the activation of beige adipocytes is beneficial to energy expenditure, the potential mechanisms for the induction of differentiation remain unclear. Recent research demonstrates that the peroxisome proliferator-activated receptor-gamma (PPAR*γ*) protein can promote the activation of beige adipocyte through activating PPAR coactivator 1-alpha (PGC-1*α*), by which UCP1 expression increases in the inner mitochondrial membrane of beige adipocyte to stimulate thermogenesis [11]. Therefore, the activation of the PPAR*γ*/PGC-1*α* pathway may be a potential target for obesity treatment. MicroRNAs (miRNAs) are endogenous noncoding single-stranded RNAs composed of 19-23 nucleotides that may play a critical regulatory role in a variety of biological processes, especially in the posttranscriptional regulation of proteins [12]. miR-27a can inhibit the expression of PPAR*γ*, acting as a negative obesity regulator [13, 14]. Therefore, we hypothesized that the antiobesity effect on inhibiting the expression of miR-27 might depend on the activation of the beige adipocytes.

Jinlida granules (JLD), a traditional Chinese medicine (TCM) prescription produced by Yiling Pharmaceutical Co., Ltd. (Shijiazhuang, Hebei Province, China) are clinically used for the treatment of type 2 diabetes. Previous studies have shown that JLD can maintain glucose and lipid homeostasis, increase insulin sensitivity, inhibit fat accumulation, and promote the expression of brown adipose cell-derived adipokine [15–20]. However, there is no study about the antiobesity effect of JLD associated with the activation of beige adipocytes. The aim of this study was to reveal the antiobesity effect of JLD and explore the potential mechanism by activating the beige adipocytes.

## 2. Materials and Methods

### 2.1. Oral Gavage Preparation

JLD, composed of 17 TCMs (ginseng, sealwort, *Rhizoma Atractylodis*, *Sophora flavescens*, *Ophiopogon japonicus*, dried rehmannia root, prepared fleece flower root, dogwood, *Herba Eupatorii, Coptis*, *Epimedium*, poria, *Anemarrhena*, lychee seed, *Salvia*, *Pueraria montana Lobata*, and *Cortex Lycii*), was purchased from Shijiazhuang Yiling Pharmaceutical Co., Ltd. (Shijiazhuang, China) and suspended in 0.5% carboxymethyl cellulose (CMC) for intragastric administration to mice at a daily dose of 3.8 g/kg for 10 weeks, as previously reported [19–22]. Rosiglitazone tablets, purchased from Chengdu Ruiheng Pharmaceutical Co., Ltd. (Chengdu, China), were ground into a powder and suspended in 0.5% CMC for intragastric administration to mice at a daily dose of 4 mg/kg.

### 2.2. Cellular Drug Preparation

JLD (3 ± 0.1 g) was dissolved in 30 ± 1 mL serum-free DMEM/F12 medium; after ultrasonic solubilization, the mixture was centrifuged at 4000 rpm and the supernatant was filtered through a 0.22 *μ*m filter (Millipore, USA).

Finally, JLD suspension with concentration of 100 ± 10 mg/mL was obtained, and the liquid was stored at -20°C.

### 2.3. Grouping and Feeding Animals

All animal studies were approved by the Ethical Review Board of Hebei Yiling Pharmaceutical Research Company (Shijiazhuang, China). Male db/db mice (4 weeks old, 20 ± 2 g) were purchased from Jiangsu Jicui Yaokang Biotechnology Co., Ltd. (Nanjing, China) and reared at 22°C, 50% humidity, and 12-hour simulated natural light with adequate diet and water. The mice were acclimatized for one week before being randomly divided into three groups (*n* = 20). The blank group was administered with CMC by oral gavage, while the control group and JLD group mice were administered with rosiglitazone and JLD, respectively, daily for 10 weeks.

The body weight of the mice was measured weekly from the fourth week onward. In the last week of the study, the mice were exposed to 4°C environment to test their ability of cold tolerance, and their anal temperatures were recorded as body temperatures. The mice were anesthetized and euthanized by cervical dislocation. The weight of subcutaneous, inguinal, epididymal adipose and liver tissues was recorded and then stored in liquid nitrogen or with a fixative for future analysis.

### 2.4. Biochemical Parameter Detection

Total triglyceride (TG), low-density lipoprotein cholesterol (LDL-C), total cholesterol (TC), serum aspartate aminotransferase (AST), alanine aminotransferase (ALT), and glucose (GLU) levels were measured by using an automated biochemical analyzer (Hitachi 7080, Japan) and a commercial kit (Jiuqiang, Beijing, China).

### 2.5. Pathology and Immunohistochemistry

The fixed, embedded, and sectioned adipose tissue and liver specimens were stained with hematoxylin and eosin (H&E). After staining, morphological tests were performed by conventional histological methods. The adipose tissue sections were deparaffinized and processed using an immunohistochemistry kit (ZSGB-BIO, China). Target proteins were labeled with two antibodies: UCP1 (10983, Abcam) and CD137 (EPR23218-111, Abcam), and fluorescence in situ hybridization (FISH) was performed with an miR-27a probe (-DIG-GCGGAACUUAGCCACUGUGAA-DIG-3′). The slides were observed and photographed by using a fluorescence microscope (Leica DM6000, Germany), and the fluorescence intensity was calculated by using ImageJ (1.53).

### 2.6. Cell Culture and Induction

The X9 cell line, a beige adipose precursor cell developed by the Harvard Medical School [9], was purchased from the ATCC Cell Bank (CRL-3282, USA) and cultured in DMEM/F12 medium (ATCC30-2006), 15% fetal bovine serum (FBS), and 2.36 mM L-alanyl-L-glutamine. The appropriate JLD concentration was screened using the MTS method. During cell induction, the blank group was incubated in DMEM/F12 medium containing 10% FBS, which was replaced every 2 days. In the control group, 5 *μ*M dexamethasone, 0.5 *μ*g/mL insulin, 0.5 mM isobutylmethylxanthine q5, 1 *μ*M rosiglitazone, and 1 nM T3 in DMEM/F12 medium containing 10% FBS were added to the cultures on the first day of induction (day 0). This solution was replaced every other day (day 2) until the DMEM/F12 medium containing 10% FBS, 0.5 *μ*g/mL insulin, and 1 nM T3 was added to the cultures (day 4). After 2 days (day 6), cell differentiation was complete, and follow-up experiments were conducted.

### 2.7. Nile Red Staining

X9 cells were seeded in 96-well plates at a density of no less than 1 × 10^5^/mL. After inducing differentiation and maturation, the medium was discarded, the cells were washed with phosphate-buffered saline, and Nile red dye was added. The cells were observed using a fluorescence microscope to examine the morphology and number of lipid droplets in the cells. The fluorescence intensity was measured using a microplate reader (BioTek, USA).

### 2.8. Confocal Microscopy

The X9 cells were seeded into 35 mm glass-bottom cell culture dishes with a density no less than 1 × 10^5^/mL and fixed after the induction of differentiation and maturation. UCP1 was observed by using immunofluorescence under a laser confocal microscope (ZEISS, Germany) and images were collected, and the fluorescence intensity was calculated by using ImageJ (1.53).

### 2.9. Western Blotting

Protein samples were collected, separated on a 12% SurePAGE preformed gel (GenScript, China), and transferred onto polyvinylidene difluoride (PVDF) membranes (Millipore, USA). The membranes were completely sealed with a sealing solution and incubated with primary antibody overnight at 4°C and then incubated with a secondary antibody for 1 h at 4°C. After incubation, an Odyssey infrared laser imaging system (LI-COR, USA) was used for signal detection. Protein quantification was performed using image analysis software. The antibodies used in this experiment include anti-PPAR*γ* (ab45036, Abcam), anti-PGC-1*α* (ab54481, Abcam), anti-UCP1 (ab10983, Abcam), anti-oxphos (ab110413, Abcam), anti-*β*-actin (3700, CST), and anti-GAPDH (5174, CST).

### 2.10. Quantitative Reverse Transcription-Polymerase Chain Reaction (qRT-PCR)

X9 cells were seeded in 6-well plates with a density no less than 1 × 10^5^/mL. After induced differentiation and maturation, total RNA was extracted by using the Eastep™ Total RNA Extraction Kit (Promega, China). cDNA was synthesized by using the Prime Script™ synthesis kit (TaKaRa, China), detected by SYBR Green PCR Master Mix (TaKaRa), and analyzed by using an ABI 7900 real-time fluorescent quantitative PCR instrument (ABI, USA). The miRNA was extracted from X9 cells on days 1, 3, and 5 after induction, and cDNA was synthesized by using the Mir-X miRNA First-Strand Synthesis Kit (Takara Bio, USA). The expression level was detected by using SYBR Green PCR Master Mix (TaKaRa) and analyzed by using the ABI 7900 real-time fluorescent quantitative PCR instrument. The primer sequence is shown in Table 1.

### 2.11. Molecular Docking Evaluation

In the previous study, nine compounds were identified in the established ultraperformance liquid chromatography (UPLC) fingerprints of JLD, including danshensu sodium, puerarin, salvianolic acid B, epmedin B, epmedin C, icariin, ginsenoside Rb1, ginsenoside Rb2, and ginsenoside Rc [23]. In order to preliminarily identify the molecules with the potential activating effect on UCP1, a molecular docking method was taken to evaluate the binding affinity. AutoDock Vina 1.1.2 was used to conduct the docking task [24]. Through literature search, we obtained five small molecular compounds (berberine [25], thiazolidinedione [26], rhein [27], formononetin [28], and fluvastatin sodium [29]), which can activate UCP1 as the control. The compound structures were downloaded from the PubChem database in SDF format [30]. Three-dimensional structures of UCP1 (P25874) were obtained from the AlphaFold database (https://alphafold.ebi.ac.uk) [31, 32], which is an openly accessible and extensive database of high-accuracy protein-structure predictions. ProteinsPLUS (https://proteins.plus/) was used to predict the active pockets. The ligands and receptors were prepared according to the tutorial of AutoDock Vina. If the docking score of compounds in JLD is higher than that of all control molecules and have good binding activity in more than one site, they are more likely to have potential activation effect on UCP1. The conformation with the lowest affinity was used as the best docking conformation, and PLIP was taken to visualize the interaction mode [33].

### 2.12. Statistics

All experimental data were expressed as the mean ± standard deviation. SPSS19.0 software was used for statistical analysis. One-way analysis of variance (ANOVA) was used for comparison among groups, and *P* < 0.05 was considered statistically significant. All graphs are done through GraphPad Prism (V.5.01).

## 3. Results

### 3.1. JLD Reduced Adipose Content and Maintained Body Temperature in db/db Mice

**Figure 1:**
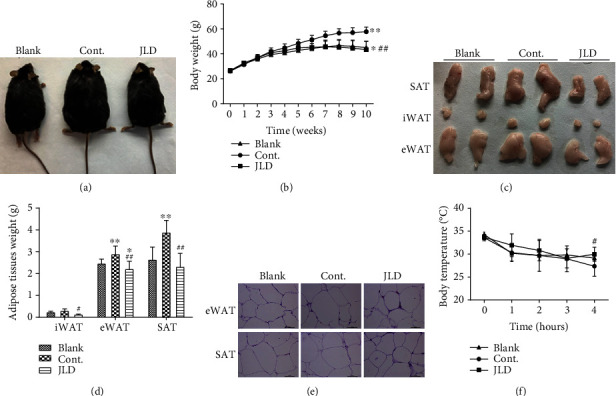
JLD reduces fat content and maintains body temperature in db/db mice. (a) Representative pictures of the weight in mice after 10 weeks of intervention and body weight-time curve (*n* = 15-20). (b) Body weight-time curve (*n* = 15-20). (c) Representative pictures of SAT, iWAT, and eWAT. (d) SAT, iWAT, and eWAT weights (*n* = 8-10). (e) Representative H&E staining of iWAT and eWAT (400x). (f) Body temperature-time curve (*n* = 5-6). Data are shown as the mean ± SD. ^∗^*P* < 0.05, ^∗∗^*P* < 0.01 vs. blank group, ^#^*P* < 0.05, ^##^*P* < 0.01 vs. control group. All graphs are done through GraphPad Prism (V.5.01).

The fat accumulation in mice can be observed intuitively through the body weight, so we observed the body shape, adipose morphology, body weight, and adipose weight of mice to understand the situation of fat accumulation and metabolism in mice. In addition, the maintenance of body temperature was also observed under 4°C environment. The results showed that JLD significantly reduced body weight in db/db mice. The body weight became different from the third week of taking JLD (Figures 1(a) and 1(b)). The mice in the control group gained weight significantly faster than the mice in the JLD-treated group, and the same results were observed in the blank group (Figure 1(b)). Epididymal white adipose tissue (eWAT) was reduced in the JLD-treated group compared with the blank group (Figures 1(c) and 1(d)). Compared with mice in the control group, JLD-treated mice had reduced subcutaneous white adipose tissue (SAT), eWAT, and inguinal subcutaneous white adipose tissue (iWAT) (Figures 1(c) and 1(d)). Additionally, compared with the control group, the morphology of fat cells in the JLD treatment group was significantly different, with more complete cell structure and smaller cell morphology (Figure 1(e)). Interestingly, the data showed that the body temperature of mice in the JLD-treated group was more stable at 4°C environment and was significantly higher than that of control mice after 4 h exposure to 4°C (Figure 1(f)). The above data suggest that JLD could reduce the lipid accumulation in mice and maintained the body temperature.

### 3.2. JLD Maintained Glucose and Lipid Homeostasis in db/db Mice

**Figure 2:**
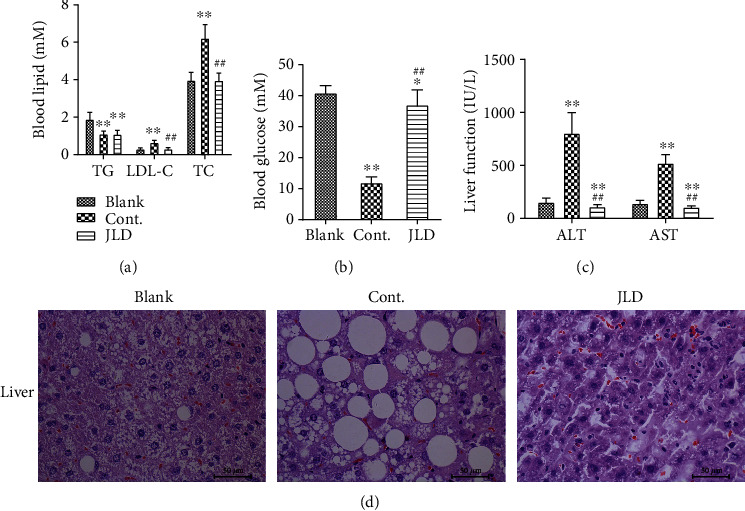
JLD maintained glucose and lipid homeostasis in db/db mice. (a, b) Fasting serum lipid levels of TG, LDL-C, TC, and GLU (*n* = 14-16). (c) Serum AST and ALT concentrations (*n* = 10-12). (d) Representative H&E staining of the liver (400x). Data are shown as the mean ± SD. ^∗^*P* < 0.05, ^∗∗^*P* < 0.01 vs. blank group, ^##^*P* < 0.01 vs. control group. All graphs are done through GraphPad Prism (V.5.01).

In order to further understand the effects of JLD on glucose and lipid metabolism, we observed the blood lipid, blood glucose, liver function, and lipid deposition in the liver of mice. Spontaneous obesity occurred in db/db mice, leading to disorders of glucose and lipid. JLD also plays an important role in improving lipid metabolism disorders. In the JLD-treated group, serum TG levels were lower than those in the blank group, and LDL-C and TC levels were significantly lower than those in the control group (Figure 2(a)). Additionally, GLU levels were significantly lower than those in the blank group (Figure 2(b)). JLD reduced liver damage caused by high lipid levels, indicated by significantly lower ALT and AST levels in the JLD-treated mice than those in the control group (Figure 2(c)). H&E staining result showed that hepatocyte ballooning in the JLD-treated group was significantly reduced compared to that in the other groups (Figure 2(d)). These results indicated that JLD could regulate glucose and lipid metabolism in db/db mice and improve liver injury caused by high lipid levels.

### 3.3. JLD Activated Beige Adipocytes in db/db Mice

**Figure 3:**
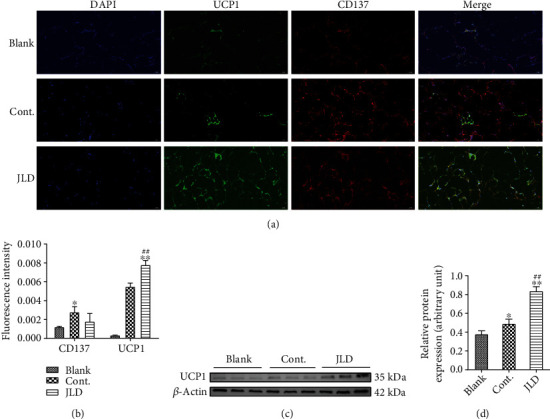
JLD activates the activity of beige adipocytes in db/db mice. (a) Fluorescence images of UCP1 and CD137 in the subcutaneous fat tissue of db/db mice (200x). (b) Fluorescence intensity of UCP1 and CD137 in the subcutaneous fat tissue of db/db mice. (c) Western blot analysis of thermogenic-related protein UCP1 expression in subcutaneous fat tissue of db/db mice and (d) quantitative measurement of relative protein expression (*n* = 4-5). Data are shown as the mean ± SD. ^∗^*P* < 0.05, ^∗∗^*P* < 0.01 vs. blank group, ^##^*P* < 0.01 vs. control group. The samples derive from the same experiment, and gels/blots were processed in parallel. Full-length gels/blots are presented in Supplementary Figure S1B. All graphs are done through GraphPad Prism (V.5.01).

**Figure 4:**
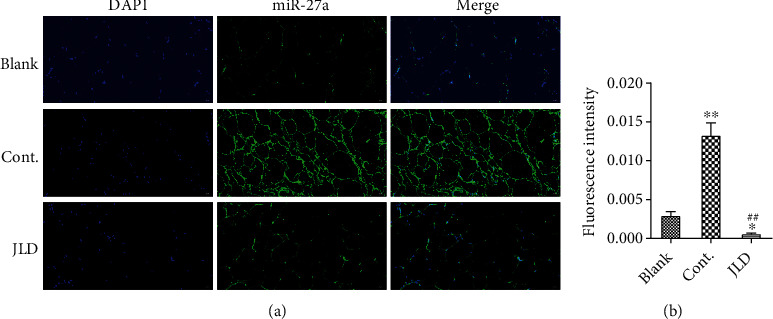
JLD inhibits the expression of miR-27a in the subcutaneous fat tissue of db/db mice. (a) Fluorescence images of miR-27a in the subcutaneous fat tissue of db/db mice (200x). (b) Fluorescence intensity of miR-27a in the subcutaneous fat tissue of db/db mice. Data are shown as the mean ± SD. ^∗^*P* < 0.05, ^∗∗^*P* < 0.01 vs. blank group, ^##^*P* < 0.01 vs. control group. All graphs are done through GraphPad Prism (V.5.01).

To understand the change of WAT after JLD treatment, we observed the activation of WAT by immunofluorescence, in situ hybridization, and western blot. However, UCP1 expression increased in WAT of db/db mice treated with JLD compared to the control group, although no increase was observed in beige adipocytes (Figures 3(a) and 3(b)), indicating that JLD induces the activity of beige adipocyte. To identify beige fat, CD137 was specifically labeled, which is a member of the tumor necrosis factor receptor superfamily and has recently been identified as one of the marker proteins of beige fat [9]. Western blotting results showed that UCP1 expression was significantly increased in the JLD-treated group compared with the control group (Figures 3(c) and 3(d), Supplementary Figure S1A). The inhibition of WAT is closely related to the expression of miRNA27a. Therefore, we further investigated the expression of miR-27a using fluorescence in situ hybridization, and the results showed that JLD inhibited miR-27a expression. The high expression of miR-27a in the control group may be due to the gain of adipose and weight (Figures 4(a) and 4(b)).

### 3.4. JLD Reduced Lipid Deposition in X9 Cells

**Figure 5:**
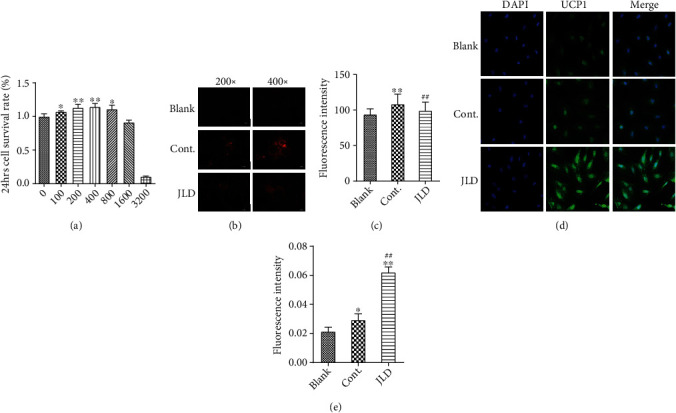
JLD reduces lipid deposition in X9 cells. (a) The correlations of JLD concentration with X9 cell density. (b) Nile red staining of blank, control, and JLD-treated X9 cells. (c) Fluorescence intensity of Nile red-stained lipid droplet. (d) Fluorescence images of UCP1 (400x) and fluorescence intensity of UCP1 in the X9 cells. (e) Fluorescence intensity of UCP1 in the X9 cells. Data are shown as the mean ± SD. ^∗^*P* < 0.05, ^∗∗^*P* < 0.01 vs. blank group, ^##^*P* < 0.01 vs. control group. All graphs are done through GraphPad Prism (V.5.01).

We verified the results of animal experiments at the cellular level to observe whether JLD could activate beige adipocytes and increase the expression of UCP1 in beige adipocytes. We used the MTS assay to examine the effect of different concentrations of JLD on the activity of X9 cells (Figure 5(a)), and 400 *μ*g/mL was selected as the dose. We found that JLD could markedly reduce the lipid droplet content (Figures 5(b) and 5(c)) and increase the expression of UCP1 in X9 cells (Figures 5(d) and 5(e)) compared with the control group. These data suggest that JLD can enhance the expression of UCP1, a thermogenesis-related protein, reducing intracellular lipid deposition.

### 3.5. JLD Inhibited the Expression of miR-27a to Promote the Activation of Beige Adipocyte

**Figure 6:**
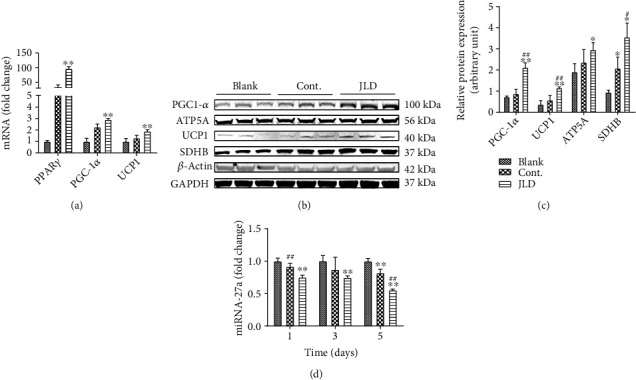
JLD inhibits the expression of miR-27a to promote the activation of beige adipocytes. (a) Western blot analysis of thermogenic-related protein UCP1 and PGC-1*α* oxidation-related protein ATP5A and SDHB expression in X9 cells treated with inducer or inducer and JLD. (b) Quantitative measurement of relative protein expression. (c) qRT-PCR analysis of thermogenic and fatty acid oxidation-related gene expression from X9 cells treated with inducer or inducer and JLD. (d) Relative expressions of miR-27a in X9 cells treated with inducer or inducer and JLD at 1, 3, and 5 days. Data are shown as the mean ± SD. ^∗^*P* < 0.05, ^∗∗^*P* < 0.01 vs. blank group, ^#^*P* < 0.05, ^##^*P* < 0.01 vs. control group. The samples derive from the same experiment, and gels/blots were processed in parallel. Full-length blots/gels are presented in Supplementary Figure S1B. All graphs are done through GraphPad Prism (V.5.01).

To understand how JLD activates beige adipose, we used western blot, PCR, and confocal microscopy methods to observe the expression of UCP1, PPAR*γ*, PGC-1*α*, and miR-27a. JLD increased mRNA expression of thermogenic genes such as PPAR*γ*, PGC-1*α*, and UCP1 (Figure 6(a)). Simultaneously, JLD also increased the expression of PGC-1*α*, UCP1, and oxidation-related proteins, ATP5A and SDHB (Figures 6(b) and 6(c), Supplementary Figure S1B), and significantly inhibited the expression of miR-27a (Figure 6(d)).

### 3.6. Small Molecules Potentially Activating UCP1 in JLD

**Figure 7:**
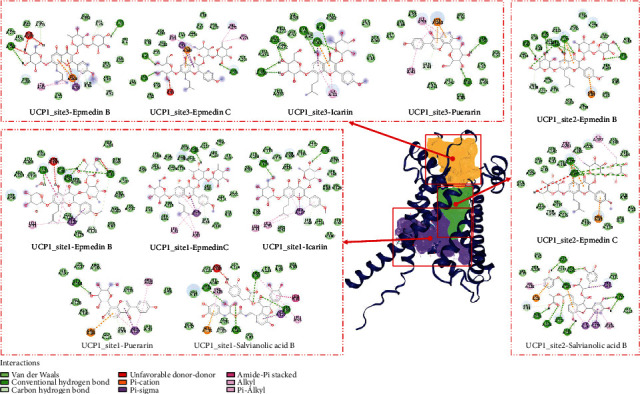
The interaction mode between five core components and three UCP1 sites. The UCP1 active sites were predicted by ProteinsPLUS. Purple is site 1 (drug score = 0.82), green is site 2 (drug score = 0.81), and yellow is site 3 (drug score = 0.8).

Based on the “drug score” obtained by ProteinsPLUS, we have chosen the top three active pockets for UCP1. The docking results of 5 control molecules and 9 compounds in JLD with three sites of UCP1 are shown in Table S1. In UCP1_site1, epmedin C and icariin got the lowest binding affinity (-8.8 kcal/mol). In UCP1_site2, salvianolic acid B obtained the lowest binding affinity (-9.3 kcal/mol). Epmedin B and epmedin C showed good binding activity at all three sites. Secondly, salvianolic acid B, icariin, and puerarin have good binding activities to more than one sites. The interaction mode between five core components and three UCP1 sites is shown in Figure 7.

## 4. Discussion

The study proved that JLD treatment significantly ameliorated HFD-induced obesity and adipose accumulation, maintained glucose and lipid homeostasis, and improved hepatic steatosis and inflammation.

Beige adipocytes, found in white adipose depots, are inducible to express the UCP1 in cold or drug stimulation [34, 35]. In contrast to white adipocytes, beige adipocytes can increase energy metabolism and reduce adipose accumulation in the activated state [36]; thus, beige adipocytes are considered therapeutic targets for the treatment of obesity and its complications, such as metabolic syndrome, diabetes, and nonalcoholic steatohepatitis.

In this study, we used db/db mice, a model of spontaneous obesity due to the leptin receptor deficiency [37], to explore the antiobesity effect of JLD. Consistent with the previous study [20], our study also confirmed this point. We also observed the alleviating effect of JLD on lipid deposition in the liver. Compelling evidence suggests that adipokines released by hypertrophic adipocytes affect multiple organs and biological processes [38], especially in chronic system inflammation and glucose metabolism dysfunction [39–42]. We found that JLD improved the glucose metabolism by reducing GLU, consistent with previous reports [16, 20]. We considered that JLD has a great potential to improve the disorders of glucose and lipid metabolism as well as concomitant inflammatory responses.

Activation of beige adipocytes is characterized by upregulated expression of UCP1, which has been identified as a thermogenic gene that releases energy in the form of heat in adipose tissue [43]. To investigate whether the antiobesity effect of JLD depended on the activation of beige adipocytes, the cold tolerance test showed that the body temperature of mice in the JLD-treated group was more stable than that of mice in other groups. Using immunofluorescence, we found a significant increase in UCP1 expression, suggesting that the increased heat release in JLD-treated mice might be due to the differentiation of beige adipocytes in the white adipose depots. To explore whether JLD plays a direct role in the differentiation of beige adipocytes, we induced the differentiation of the beige adipocyte precursor (X9 cell line) in vitro, and JLD intervention was given in this process [9, 44, 45]. The result showed that JLD increased the thermogenesis of beige adipocytes. In addition, we found that JLD also increased the expression level of ATP5A and SDHB protein, which may promote the energy metabolism through oxidative phosphorylation. This suggests that JLD can activate beige adipocytes and increase their energy metabolism.

Previous studies have shown that miR-27a could inhibit the expression of PPAR*γ*, which promotes the differentiation of beige adipocytes in the white adipose depots [13, 46, 47]. The prediction assay for the miRNAs binding to the PPAR*γ* mRNA also confirmed the binding site of miR-27a. Therefore, we performed fluorescence in situ hybridization on mouse adipose sections and found that miR-27a expression in the JLD-treated group was lower than that in untreated mice. In subsequent qPCR assays, we revealed that JLD inhibited the expression level of miR-27a in the postdifferentiated X9 cells. All these results indicate that the potential mechanism of JLD-induced activation of beige adipocytes may depend on inhibiting miR27a expression, which could inhibit PPAR*γ* expression, to activate beige fat.

Through molecular docking, we obtained five potential candidate compounds in JLD with potential activation effect on UCP1. The binding affinity between icariin and UCP1 sites 1 and 3 was -8.8 and -7.4 kcal/mol, respectively, indicating good potential activity. One study showed that icariin can increase UCP1 expression in white adipocytes by increasing the expression of PGC-1*α* [48]. The binding affinity between salvianolic acid B and UCP1 sites 1 and 2 was -8.6 and -9.3 kcal/mol, respectively. And it may be involved in lipid metabolism and energy metabolism in mice, playing an antiobesity role by regulated energy metabolism [49]. Puerarin can increase the expression of PPAR*γ* in bovine preadipocytes [50]. In this study, puerarin showed good binding activity with UCP1, with binding affinity of -7.9 and -7.8 kcal/mol to site 1 and site 2, respectively. Although ginsenoside Rb1, ginsenoside Rb2, and ginsenoside Rc were not screened by the strict screening criteria, they still had certain binding activity on UCP1 sites 1 or 2. Ginsenoside Rb1 can promote browning effect by enhancing protein expression of PR domain containing 16 (PRDM16), PGC-1*α*, and UCP1 [51]. Ginsenoside Rb2 can ameliorate obesity and metabolic disorders by inducing gene expression of PGC-1*α* and UCP1 [52]. In addition, ginsenoside Rc can resist obesity by inhibiting the expression of PPAR*γ* and CCAAT/enhancer binding protein (C/EBP) [53]. Notably, no association between epmedin B or epmedin C and obesity, metabolic disorder, or UCP1 targets has been reported. However, the results showed that epmedin B and epmedin C had good binding affinity for all three UCP1 sites, indicating good verifiability. In short, JLD activates the beige adipocytes through its multiple active ingredients, and further experiments are needed to confirm the activity.

## 5. Conclusions

Our data show that JLD has an antiobesity effect in the db/db mouse model of spontaneous obesity, while improving maintained glucose and lipid homeostasis and hepatic steatosis. This study for the first time demonstrates that JLD may induce the activation of beige adipocytes by suppressing the expression of miR-27a. Epmedin B, epmedin C, icariin, puerarin, and salvianolic acid B may be the pharmacodynamic material basis of JLD that plays an important role in regulating UCP1. These findings suggest that JLD has great potential in maintained glucose and lipid homeostasis, making it useful for controlling the body weight and alleviating the glucose and lipid homeostasis dysfunction caused by obesity.

## Figures and Tables

**Table 1:** Primer sequences used for qRT-PCR in this study.

Primer	Forward	Reverse
PPAR*γ*	CCTGGACCTCTGCTGGTGAT	GCTGGAGAAATCAACCGTGG
PGC-1*α*	GGCACCTGAACAGAACGAAC	CAACAGGCATCAGCAGTGTC
UCP1	ACGTCCCCTGCCATTTACTG	CCCTTTGAAAAAGGCCGTCG
GAPDH	CTGCGACTTCAACAGCAACT	GAGTTGGGATAGGGCCTCTC
miRNA27a	TGCGCTTCACAGTGGCTAAGT	CCAGTGCAGGGTCCGAGGTATT
U6	CGCTTCGGCAGCACATATAC	AAATATGGAACGCTTCACGA

## Data Availability

The data used to support the findings of this study are included within the article.

## Abbreviations

JLD: Jinlida granules
PCR: Polymerase chain reaction
UCP1: Uncoupling protein 1
WAT: White adipose tissue
PPAR*γ*: Peroxisome proliferator-activated receptor-gamma
PGC-1*α*: PPAR coactivator 1-alpha
TCM: Traditional Chinese medicine
TG: Total triglyceride
LDL-C: Low-density lipoprotein cholesterol
TC: Total cholesterol
AST: Serum aspartate aminotransferase
ALT: Alanine aminotransferase
GLU: Glucose
eWAT: Epididymal white adipose tissue
iWAT: Inguinal subcutaneous white adipose tissue
PRDM16: PR domain containing 16
C/EBP: CCAAT/enhancer binding protein.

## Supplementary Materials

Supplementary Materials Table S1: docking scores of 5 control molecules and 9 compounds in JLD with three active sites of UCP1. Fig S1_raw_images.
