# Supplementary material for: Jinlida Granules Reduce Obesity in db/db Mice by Activating Beige Adipocytes
Source: Biomed Res Int. 2022 May 19;2022:4483009. doi: 10.1155/2022/4483009 (PMC9135524; doi:10.1155/2022/4483009)
Supplement: Supplementary Materials — Table S1: docking scores of 5 control molecules and 9 compounds in JLD with three active sites of UCP1. Fig S1_raw_images. [file 4483009.f1.pdf]

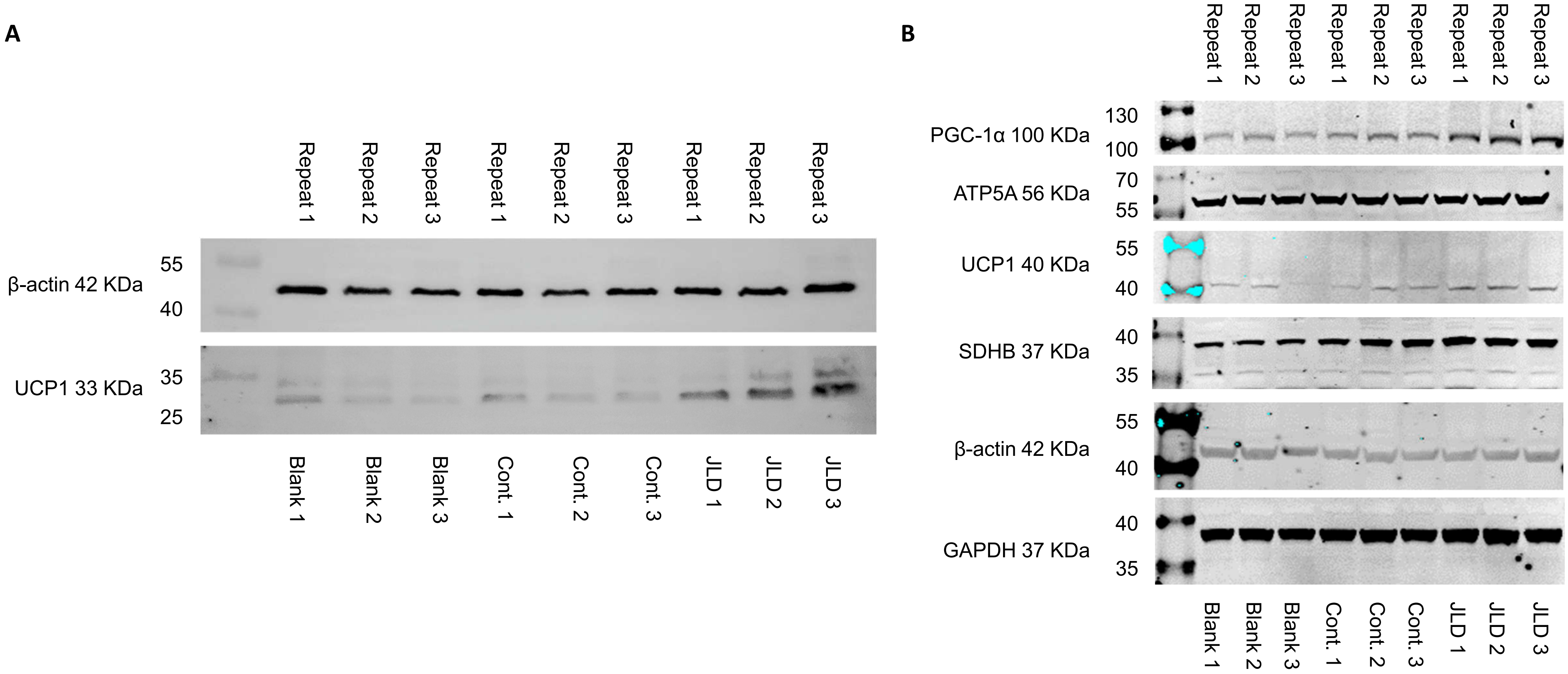

S1_raw_images. (A) The whole blot after cutting membrane at molecular weight 40~55 kDa and 25~35 kDa for β-actin (42 kDa) and UCP1 (33 kDa) respectively. (B) The whole blot after cutting membrane at molecular weight 100~130 kDa, 55~70kDa, 40~55 kDa, 40~55 kDa, 35~40 kDa, and 35~40 kDa for PGC-1α (100 kDa), ATP5A (56 kDa), UCP1 (40kDa), SDHB (37 kDa), β-actin (42 kDa) and GAPDH (37 kDa) respectively.
